# Editorial Special Issue 2022

**DOI:** 10.1007/s41125-023-00089-z

**Published:** 2023-03-20

**Authors:** Rahel M. Schomaker, Stoyan Panov

**Affiliations:** 1 CUAS Villach, Austria/FÖV Speyer, Germany; 2 Albert-Ludwigs-Universität Freiburg, Freiburg, Germany

## European Journal for Security Research (2023)

While the Covid-19 pandemic has abated in the past months, without being completely over yet for many countries worldwide, new challenges like the war in Ukraine and the related migration movements, resource shortages and natural disasters have emerged in this period, affecting public institutions as well as societies and markets. Scrutinizing the resulting challenges, security research comprises even more themes, dimensions and dynamics than before—not only legal, military or technical aspects, but also the social and organizational dimension of security comes to the fore in such situations.

This special issue on the Covid-19 pandemic is devoted to aspects and topics that typically do not stand in the main focus of security research, brushing on the blurring lines between the individual, the society and the state, the public and the private sphere. This focus does not only provide innovative insights into legal, organizational and social aspects of the Covid-19 pandemic. It also may induce more interdisciplinary research on the ongoing multi-faced crises, their origins, consequences and potential solutions.

While surely a single issue of a scientific journal is one piece of the puzzle, we would like to thank the authors and reviewers of this special issue for their inspiring contributions. This special issue would have not been possible without the kind support and encouragement of Prof. Stefan Kaufmann, Editor-in-Chief of the journal, to whom goes our sincere professional appreciation and personal gratitude.

The first article in this special issue by Malte Schönefeld, Patricia M. Schütte, Yannic Schulte and Frank Fiedrich focuses on a selected major business sector, the event sector in Germany, scrutinizing the stop-and-go procedures of re-opening the sector during the pandemic with a particular eye on the social impact. The case study carves out in how, particularly due to the characteristics of the federal system in Germany, convincing re-opening concepts were difficult to identify.

The explorative study provided by Florian Roth, Benjamin Kaluza, Katharina Pfeffer, Esther Rümelin, Joel Kirchner, Maike Overmeyer, Florian Neisser, Thomas Jackwerth-Rice, Aleyna Kilicaslan and Johannes Sautter provides insights in the learning and innovativeness of civil protection organizations in the Covid-19 pandemic, in particular identifying factors for such organizations to shape crisis-related learning processes. Drawing to semi-structured interviews with civil protection experts from Switzerland, Austria, Germany, Sweden and Italy, speed and flexibility of crisis response, as well as solid collaborations in networks and the need for digital capabilities and infrastructures are identified as main success factors. The finding is unequivocal: the civil protection organizations need to respond quickly and adapt correspondingly. Civil protection organizations are hopefully more resilient now, building on their innovative collaboration during the crisis. One essential lesson is the need for flexibility and adaptability in inter-crisis mode of reaction and learning.

The third article by Nils Lüttschwager focuses on psychosocial consequences for the population, analyzing the role of psychosocial consequences within the work of German crisis management organizations during the pandemic. Based on interviews with decision-makers, e.g. from municipal and state administrations, public health departments, and aid organizations, the author finds that while crisis managers perceive problems related to the psychosocial situation as relevant, but in a very selective and somewhat negligent way. Given the ongoing crises that hit the politico-administrative systems in Europe, these results not only provide leeway for further research, but also deliver important insights for German public managers.

Andreas Langenohl touches upon another angle of coping with the pandemic in the public sphere, arguing that government responses to the pandemic relied more on „biopolitical governance of populations“, but only in a limited way on the „symbolic governance of public spheres“. Drawing to policy documents and their medial representation, the author finds that the politics of pandemic security focused on the regulation of population aggregates and movements, but paid no attention to non-stochastic social aggregates, even if these aggregates produced mobilization against the politics of pandemic governance in many liberal democracies, what can be understood as an insistence on sovereign power.

In this line, Pascal Berger focuses on the fact that the measures taken to contain the pandemic curtailed the fundamental rights of citizens in many countries; he analyzes the role of courts and the principle of proportionality where facts and normativity meet in the German case. With a view on polarization trends, the selection and interpretation of scientific expertise itself is a politicised value, and court rulings confirm and reproduce a political strategy that shows a balance of interests with a priority of life and health over other liberties.

